# Comment on Manzoor, H. et al. Efficacy of Different Dosing Regimens of IgE Targeted Biologic Omalizumab for Chronic Spontaneous Urticaria in Adult and Pediatric Populations: A Meta-Analysis. *Healthcare* 2022, *10*, 2579

**DOI:** 10.3390/healthcare12050553

**Published:** 2024-02-28

**Authors:** Amin Tajerian

**Affiliations:** School of Medicine, Arak University of Medical Sciences, Arak 3819693345, Iran; a.tajeriyan@arakmu.ac.ir

In December 2022, the paper “Efficacy of Different Dosing Regimens of IgE Targeted Biologic Omalizumab for Chronic Spontaneous Urticaria in Adult and Pediatric Populations: A Meta-Analysis” was published in the *Healthcare* journal. This meta-analysis on chronic spontaneous urticaria assesses the efficacy of different omalizumab dosages based on weekly itching, wheal scores, UAS7, and response rates. Results suggest that both 150 mg and 300 mg doses exhibit significant improvements, indicating omalizumab is an effective intervention for individuals unresponsive to numerous therapies, particularly high-dose H1-antihistamines [1].

The X-ACT trial, conducted by Staubach et al., yielded findings published in two distinct articles in 2016 [2] and 2018 [3], contrary to the assertion made by Manzoor et al. that the findings were published in 2015. The trial by Staubach et al. had 44 patients in the omalizumab group and 47 patients in the placebo group and did not report the proportion of responders to omalizumab, which was the criterion for inclusion in Figure 11. However, Manzoor et al. reported 252 patients in the omalizumab group and 83 patients in the placebo group for this trial, which are exactly the same numbers in Kaplan et al.’s 2013 [4] trial. This discrepancy raises significant concerns about the reliability of Manzoor et al.’s analysis.

Moreover, this mistake has led to a change in the overall magnitude of the odds ratio, given that this study involves the highest number of patients and contributes 14.6% to the final effect size. Considering that the findings of the Staubach et al. study replicate these results, together they constitute 29.2% of the effect-size weight, thereby compromising the overall coherence and accuracy of the reported findings.

The wheal score and itch score information provided in the Serrano-Candelas 2017 study deviates from the original data presentation by representing medians with Q1 and Q3, while the study in question alters these figures to mean and standard deviation (SD). It is imperative for the study to detail the formula used for this conversion [5].

The Bi et al. study titled “Adjunct therapy with probiotics for chronic urticaria in children: randomised placebo-controlled trial” investigates the effectiveness of the probiotic combination Yimingjia^®^ as an adjunct therapy for children with chronic urticaria, revealing a significant improvement in symptom scores and overall response rate compared to a placebo group. There is no mention of omalizumab intervention in this study, suggesting a potential error in referencing or inclusion. Additionally, the data presented in the tables do not align with the information associated with this study under trial number NCT03328897 [6].

In the XCUISITE study conducted by Maurer et al. in 2011, the focus was on investigating the efficacy of omalizumab in patients with chronic spontaneous urticaria (CSU) who exhibited IgE autoantibodies against thyroperoxidase (TPO). This randomized, double-blind study spanned 24 weeks, during which patients were administered subcutaneous injections of either omalizumab (dose range: 75–375 mg, determined by using the approved asthma dosing table) or a placebo. Notably, the dosing regimen did not adhere to a fixed dose of 150 or 300 mg; instead, each participant received a unique dose [7].

However, Manzor et al. did not appropriately account for this variability in dosing when presenting their findings. Figure 11 in Manzor et al.’s work divides the XCUISITE study into three groups (OMA300, OMA150, and placebo), which is inconsistent with the original study’s design. Additionally, in Figure 8, Manzor et al. erroneously assigned all 27 patients to the OMA75 group. Furthermore, in Figure 9, they incorrectly assigned all 27 patients to the OMA300 group. This misallocation of patients in both figures deviates from the actual dosing structure of the XCUISITE study [7].

It is important to note that Manzor et al. failed to consider the individualized dosing approach employed in the XCUISITE study. The figures incorrectly group patients into fixed-dose categories, leading to a misrepresentation of the original study’s methodology. Notably, the basis for assigning only 8 out of 27 XCUISITE study patients to the OMA300 group in Figure 7 is unclear and lacks transparency [7].

These inconsistencies in the data analysis could impact the overall interpretation of the study and subsequently influence clinical decisions. It is crucial to address these discrepancies to ensure the accuracy and reliability of the findings presented. Further clarification or a corrigendum from the authors regarding the identified discrepancies would be greatly appreciated by the scientific community.

It is important to note that these adjustments are not intended to discredit the study’s significance but to fortify its reliability, thereby better serving the scientific and medical community. Rectifying these issues will not only ensure the accuracy of the study but also help healthcare professionals make informed decisions for patients suffering from chronic spontaneous urticaria.
